# RNA-Seq Identifies SNP Markers for Growth Traits in Rainbow Trout

**DOI:** 10.1371/journal.pone.0036264

**Published:** 2012-05-04

**Authors:** Mohamed Salem, Roger L. Vallejo, Timothy D. Leeds, Yniv Palti, Sixin Liu, Annas Sabbagh, Caird E. Rexroad, Jianbo Yao

**Affiliations:** 1 Laboratory of Animal Biotechnology and Genomics, Division of Animal and Nutritional Sciences, West Virginia University, Morgantown, West Virginia, United States of America; 2 National Center for Cool and Cold Water Aquaculture, Kearneysville, West Virginia, United States of America; Auburn University, United States of America

## Abstract

Fast growth is an important and highly desired trait, which affects the profitability of food animal production, with feed costs accounting for the largest proportion of production costs. Traditional phenotype-based selection is typically used to select for growth traits; however, genetic improvement is slow over generations. Single nucleotide polymorphisms (SNPs) explain 90% of the genetic differences between individuals; therefore, they are most suitable for genetic evaluation and strategies that employ molecular genetics for selective breeding. SNPs found within or near a coding sequence are of particular interest because they are more likely to alter the biological function of a protein. We aimed to use SNPs to identify markers and genes associated with genetic variation in growth. RNA-Seq whole-transcriptome analysis of pooled cDNA samples from a population of rainbow trout selected for improved growth versus unselected genetic cohorts (10 fish from 1 full-sib family each) identified SNP markers associated with growth-rate. The allelic imbalances (the ratio between the allele frequencies of the fast growing sample and that of the slow growing sample) were considered at scores >5.0 as an amplification and <0.2 as loss of heterozygosity. A subset of SNPs (n = 54) were validated and evaluated for association with growth traits in 778 individuals of a three-generation parent/offspring panel representing 40 families. Twenty-two SNP markers and one mitochondrial haplotype were significantly associated with growth traits. Polymorphism of 48 of the markers was confirmed in other commercially important aquaculture stocks. Many markers were clustered into genes of metabolic energy production pathways and are suitable candidates for genetic selection. The study demonstrates that RNA-Seq at low sequence coverage of divergent populations is a fast and effective means of identifying SNPs, with allelic imbalances between phenotypes. This technique is suitable for marker development in non-model species lacking complete and well-annotated genome reference sequences.

## Introduction

Traits associated with fast and efficient growth have a major influence on the profitability of food animal production including aquaculture species. In addition, optimizing genetic×diet interactions to improve feed efficiency has the potential to reduce aquaculture effluents leading to more environmentally sustainable production. Successful selection for optimal growth rate or body weight is a key objective in aquaculture breeding programs. Traditional phenotype-based selection is typically used to select for growth traits, however, it requires several generations to optimize genetic improvement. In addition, insight into the genetic bases of growth can be used to make better selection decisions. Molecular genetics have been used to identify several genes and markers associated with quantitative traits including genetic variation explaining phenotypic differences in growth [1], [2].

Genetic maps characterizing the inheritance patterns of traits and markers have been developed and used for a wide range of species, including fish. Genetic maps are used to target discovery of allelic variation affecting traits with the ultimate goal of identifying DNA sequences underlying phenotypes [3]. Markers used in genetic mapping have been identified by a range of molecular techniques such as RFLPs, RAPDs, AFLP, microsatellites etc. However, these markers are cumbersome to use in high throughput genotyping protocols because they exist in low density and may not be widely and evenly distributed in the genome. Alternatively, SNPs are abundant and distributed widely and evenly throughout the genome. It is estimated that 90% of the genetic variation in human arises from SNPs and 4–5 SNPs for every 1000 base pairs translate to 10,000,000 points of variation [4]. SNPs are co-dominantly inherited, and highly adaptable to large-scale automated genotyping. Therefore, they are most suitable for genome-scan association studies [5]. SNPs found within or near coding sequences, called cSNPs [4], are of particular interest because they are expected to modify the function of a protein. In addition, crossing over is less likely to separate cSNP markers from genes compared to non-cSNPs. Therefore, this class of SNPs is especially useful for species without complete genome sequences/assemblies such as aquaculture species. SNPs serve as suitable markers for mapping, in comparative genome studies and in marker assisted selection (MAS) of important traits [5], [6].

Recent developments of the next-generation sequencing technologies have allowed whole transcriptomes sequencing (RNA-Seq) and SNPs discovery at fast, accurate and affordable scale. This study tests the hypotheses if RNA-Seq can be used in a non-model species to discover true SNPs with allelic imbalances between two phenotypes in a selection population, and identify genetic markers and candidate genes suitable for association studies.

Rainbow trout is one of the most intensively studied fishes in all research areas and is the most cultivated cold freshwater fish in the US [7]. It is a member of the Salmonidae family, which has been widely introduced around the world. Considerable biological knowledge has been developed for this species as a result of their widespread cultivation as a food and sport fish [8]. More is known about the physiology and biology of rainbow trout than any other fish species, and it serves extensively as a research model for other economically important fish such as Atlantic and Pacific salmon and char species [7]. The increased interest in using genomics tools for salmonids research and development is reflected in the considerable accumulation of genomic resources for rainbow trout in the last few years [9].

The molecular and genetic basis of growth traits is inadequately understood in fish [10]. A family-based selection program was initiated in 2002 at the USDA, ARS, National Center for Cool and Cold Water Aquaculture (NCCCWA) to improve growth performance in rainbow trout using traditional genetic selection. The objective of the current study was high-throughput discovery of growth-associated cSNP markers using next-generation sequencing of the whole transcriptome, RNA-Seq, and validation and characterization of these markers in a population of rainbow trout from the NCCCWA selection program. This study examined the association of these markers with variation in growth rate in a three-generation parent/offspring panel of rainbow trout. The study identified markers and genes associated with genetic variation in growth traits.

## Methods

### Ethics Statement

Institutional Animal Care and Use Committee of The United States Department of Agriculture, National Center for Cool and Cold Water Aquaculture (Leetown, WV) specifically reviewed and approved all husbandry practices used in this study (IACUC approval #056).

### Growth-selected Line and Fish Population

This study was carried out using fish from a growth-selected line at NCCCWA breeding program [11]. Briefly, this synthetic line was developed beginning in 2002 by crossbreeding 7 founder strains with known genetic diversity and domestication history. The main strains that contributed to the population were 1) University of Washington, Donaldson; 2) Kamloops/Puget Sound Steelhead cross; 3) College of Southern Idaho, House Creek; and 4) Ennis NFH Shasta strain. The growth synthetic line is a 2-yr-old winter/spring-spawning population that became a closed population in 2004 and since that has gone through 3 generations of merit-based genetic selection.

Each generation, full-sib families were produced from single-sire×single-dam matings. Eggs were reared in spring water, and water temperatures were manipulated between approximately 7 and 13°C to synchronize hatch times. Each family was stocked separately in 200-L tanks at a density of approximately 600 alevins/tank and fish were hand-fed a commercial fishmeal-based diet (50 to 55% protein, 15% fat; Ziegler Bros Inc., Gardners, PA) beginning at swim-up. Fish were randomly culled every month to maintain stocking densities <50 kg/m^3^. Neomales were developed from a subset of alevins from the 2008 year class by feeding 2 mg/kg of 17α-methyltestosterone (Sigma-Aldrich, St. Louis, MO) for 60 d post swim-up, and the masculinized females were used as sires for the following generation. At about 5-months old, fish were anesthetized using 100 mg/L of tricaine methanesulfonate (Tricaine-S, Western Chemical, Ferndale, WA) and uniquely tagged by inserting a passive integrated transponder (Avid Identification Systems Inc., Norco, CA) into the dorsal musculature, and tagged fish were combined and reared in 1,000-L communal tanks using flow-through spring water (ambient temperature ∼12.5–13.7°C). Fish were fed a commercial fishmeal-based diet (42% protein, 16% fat; Ziegler Bros Inc., Gardners, PA) using automatic feeders (Arvotec, Huutokoski, Finland). Initially, young fish were fed at a daily feeding rate ∼ 2.5% of body weight (BW), which later was gradually reduced to approximately 0.75% of BW.

Individual BW were measured at four ages, approximately 6 (Weight1), 7 (Weight2), 9 (Weight3) and 12 (Weight4) months post-hatching, each generation using a Biomark tagging station (Biomark, Boise, ID). Fish from three generations were included in this study; breeding years, 2006, 2008 and 2010. An index of 10-mo BW estimated breeding value (EBV) and thermal growth coefficient TGC EBV was used as selection criterion in the 2008 and 2010 generations, whereas the 10-mo BW EBV was used as selection criterion in the 2006 generation using MTDFREML [12]. Each generation, mating decisions were made to maximize genetic gain while constraining the rate of inbreeding to ≤1% per generation using EVA evolutionary algorithm [13]. Data from masculinized fish were removed from the growth analysis dataset.

### RNA-Seq

RNA-Seq and SNP discovery were carried out using muscle tissues collected from 10 fast growing (Average weight at ∼14 months, 1,078.6 g, SD = 87.9 g) and 10 slow growing (Average weight 643.7 g, SD 147.4 g) female rainbow trout. Each group represents a full-sib family from the 2008 hatching year of the above-mentioned growth-selected line from the NCCCWA breeding program. Tissues were flash frozen in liquid nitrogen, shipped on dry ice to WVU, then stored at −80°C until total RNA isolation. Total RNA was isolated from each sample using TRIzol™ (Invitrogen, Carlsbad, CA). Equal masses of total RNA from the samples of each group were pooled and used for RNA-Seq sequencing.

#### Sequencing, data processing and RNA-Seq analysis

cDNA libraries were prepared and sequenced on an Illumina Genome Analyzer (single-end, 36 bp read length) at the National Center for Genome Resources (Santa Fe, New Mexico) as previously described [14]. Alpheus sequence variant detection pipeline [15] was used to map sequence reads to a reference transcriptome that we previously sequenced and assembled from a double-haploid rainbow trout fish [16]. The default settings in Alpheus were used for read mapping. The SNP detection stringency conditions include at least 4 reads calling the variant, >20% reads calling the variant and >20 Quality score [17]. Putative SNPs assumed to be associated with fast growth were considered at allelic imbalances scores (the ratio between the allelic frequencies of the fast growing fish sample and that of the slow growing fish sample) >5.0 as an amplification and <0.2 as loss of heterozygosity [18].

#### Marker validation and genotyping

Putative SNPs identified by RNA-Seq as presumably associated with growth traits were initially validated by individually genotyping fish of the discovery panel (fast versus slow growing fish, 10 fish from each group as described above) using the Sequenom iPLEX Genotyping platform. Association of markers with growth traits were further estimated by genotyping 778 individuals of the growth-selected line at NCCCWA; 661 fish from hatch year 2010 belonging to 40 families (all available 38 full-sib [with no maternal or paternal relationship] and 2 paternal half-sib families) in addition to fish parents (78 fish from hatch year 2008) and grandparents (39 fish from hatch year 2006).

### Statistical Analysis of Markers’ Associations with Growth Traits

Body weight was recorded on each animal at approximately 6, 7, 9 and 12 months post-hatching months post-hatching. The specific growth rate (SGR) was estimated as:
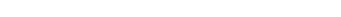
Where evaluation times were 1 = 170 d, 2 = 218 d, 3 = 282 d and 4 = 372 d post-hatching; BW is body weight; and Ln is the natural logarithm.

#### Model selection and normality test

Before performing association analysis, variables with significant contribution to the predictive power of growth trait models (Weight1-4), were identified by performing multivariable regression analysis using two linear models that included random family, fixed effect tank, and continuous covariates (age; and founder-strain composition Shasta [SH], Troutlodge [TL] and University of Washington [UW]) using STEPWISE model selection with SAS procedure REG [19]. Model A included all 6 variables (family, tank, age, SH, TL and UW); and Model B included tank, age, SH, TL and UW. The STEPWISE model selection was performed using only YC2010 offspring (n = 1657 animals) from the growth selected families (i.e., growth selection line). Whenever possible, we included the random family, fixed effect tank and covariates age and founder-strain composition SH in the linear model of association analysis to [20] minimize the variance in the sampled population; [21] to account in the association analysis for the effects of variables that have significant contribution to the predictive power of growth trait models; and [22] to ensure that the association signals detected here were mainly due to marker effects.

Growth traits (Weight1-4) were tested for departure from multivariate normal (MVN) distribution and estimated basic statistical measures for the response variables using SAS procedure UNIVARIATE [19].

#### Association analysis of nuclear SNPs with growth

From the YC2010 progeny of 40 growth selected families with their corresponding parents and grandparents (n = 1657 animals), a subset of offspring (∼17 siblings/family) were random selected (including their parents and grandparents; total of n = 877 animals) to genotype with the validated nuclear SNPs. The marker genotype data from the validated SNPs that had a genotype completion rate <0.70 and had monomorphic alleles were filtered out. After filtering out poor quality marker genotype data, we had a marker genotype dataset with 30 validated nuclear SNPs, which was used in association analysis with growth traits.

Three different algorithms of family-based association analysis were used in the association analysis of the validated nuclear SNPs with growth traits. The rate of false positive claims of association was reduced by following up the initial analysis with different methods of association analysis. Family-based association analysis methods were used to detect association signals that are robust to population stratification [21]. First, we performed family-based association analysis with PLINK version 1.07 [22]. The *t*-statistic for regression of phenotype on allele count (*b_y.x_*), and the asymptotic *P*-value for the *t*-statistic were estimated; and the empirical *P*-value was estimated using 20,000 permutations. The association results from PLINK should be taken with caution because variables (tank, age and SH) with significant contribution to the predictive power of growth trait models were not accounted in the association analysis. The current version of PLINK has a limitation of not allowing the inclusion of fixed effects and covariates in the linear model of family-based association analysis when using continuous quantitative traits (“qfam” function).

Second, we performed a family-based association analysis using the R package genome-wide association analysis with family data (GWAF) [23]. We estimated the asymptotic *P*-value for the test statistic distributed as χ^2^ with 1 and 2 DF for dominant and general model, respectively, and the proportion of phenotypic variance explained by each SNP 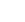. The covariates age and SH and the fixed effect tank were included in the linear model of association analysis for all growth traits (Weight2-4). Third, family-based quantitative trait linkage disequilibrium (QTLD), Bayesian quantitative trait nucleotide (BQTN) and quantitative trait disequilibrium QTDT analyses were performed with SOLAR version 4.0 [21], [24]. The BQTN association analysis was performed using SNP genotypes and haplotypes; the haplotypes were estimated with SIMWALK2, which is called internally by SOLAR. The BQTN method of association analysis can be very powerful and useful to identify genetic variants that have functional significance given a comprehensive list of SNPs in well-chosen candidate genes [25], [26].

#### Association analysis of mitochondrial SNPs with growth

The same sample of n = 877 animals used above in the association analysis of nuclear SNPs was genotyped with the validated mitochondrial SNPs. After filtering out, as indicated above, mitochondrial SNPs with poor quality marker genotype data, we had a marker genotype dataset of 24 validated mitochondrial SNPs genotyped on n = 877 animals (40 families each with ∼17 siblings). From this marker genotype dataset, a sibling was random sampled from each family to generate a population-based sample of n = 40 unrelated individuals; the random sampling was repeated to develop three sets of unrelated individuals. These three sets of unrelated individuals were used in association analysis of mitochondrial SNPs with growth traits.

Population-based association analysis was performed using the validated mitochondrial SNPs marker genotype and haplotype data with PLINK version 1.07 [22]. We estimated the *t*-statistic for regression of phenotype on allele count (*b_y.x_*); the square of the multiple correlation coefficient (*R^2^*) which measures the proportion of total variation explained by the regression *b_y.x_*; and the asymptotic *P*-value for the *t*-statistic. The empirical *P*-value was estimated using 20,000 and 10,000 permutations when using marker genotype and haplotype data in association analysis, respectively. The false discovery rate (FDR-BH) for the association analysis with marker genotype data was estimated according to Benjamini and Hochberg [27]. The results of this population-based association analysis with mitochondrial SNPs should be taken with caution because (1) the current version of PLINK has a limitation of not allowing to include fixed effects (tank) and covariates (age and SH) in the linear model of association analysis when using continuous quantitative traits; and (2) population stratification and cryptic relatedness are confounding factors in population-based association analyses which can inflate the false-positive rate [28].

### Genomic DNA Isolation for Genotyping

Genomic DNA used in genotyping samples was purified from fin clips using a modified salt-out extraction protocol. Fin clips were placed in microtubes with 500 µL of lysis buffer (10 mM Tris HC1, pH 8.0, 10 mM EDTA, 100 mM NaC1), containing 0.2% SDS and 6 µL of 10 mg/mL of proteinase K and 5 µL DTT (1 M). Tubes were incubated immediately in a water bath at 56°C for 3 h or 37°C overnight, shaking occasionally. Samples were spun for 2 min at 14,000 rpm. Supernatant was transferred to a new 1.5 mL tube. Then, 200 µL of 5 M NaCl was added to each sample before being centrifuged for 10 min at 14000 rpm. The supernatant was transferred to new microtubes where the DNA was precipitated with 1 mL of cold absolute ethanol and incubated at -20°C for 10 min. The DNA samples were centrifuged, washed with 700-µL 70% ethanol and re-suspended in 60 µl of nuclease-free water. Fish were genotyped using the Sequenom iPLEX service at Partners HealthCare Center for Personalized Genetic Medicine (PCPGM), Cambridge, MA as previously described [29].

### Linkage Analysis and Genetic Mapping

Four full-sib NCCCWA mapping families each with 46 progeny were genotyped with the 30 validated nuclear SNP markers and 214 microsatellite markers selected from the rainbow trout reference genetic maps [30]. The genotype data were checked for inconsistencies with Mendelian inheritance using PEDCHECK [31]. Linkage maps were constructed with software MULTIMAP [32]. Markers were assigned into linkage groups with the parameters of LOD ≥4 and recombination fraction r ≤0.3. The framework map for each linkage group was constructed with default parameters, and markers were added to the comprehensive map by lowering the LOD threshold one integer at a time and starting with the previous order. The linkage groups were assigned to chromosomes based on the chromosomal locations of microsatellite markers on the latest reference genetic map of rainbow trout [32].

Mitochondrial markers were positioned on the rainbow trout mitochondrial genome by aligning the flanking sequences of SNP markers against the rainbow trout mitochondrial reference sequence (GenBank: L29771.1) [33].

### Aquaculture Broodstocks

Markers’ polymorphism was assessed in unrelated fish collected from three aquaculture breeding programs: two broodstocks from the USDA-ARS Hagerman Fish Culture Experiment Station, Hagerman, ID (2009 and 2010 odd and even brood-year), two stocks from Clear Springs Foods, Buhl, ID (2009 and 2010 odd and even brood-year) and 8 stocks from Troutlodge Inc., Sumner, WA (4 spawning time broodstocks x 2 year cycles). A total of 96 unrelated fish (8 fish/broodstock) were genotyped. These samples represent populations from the main rainbow trout breeders in the US.

## Results and Discussion

### Detecting SNP Variants in Pooled Transcriptome Samples by RNA-Seq

**Figure 1:**
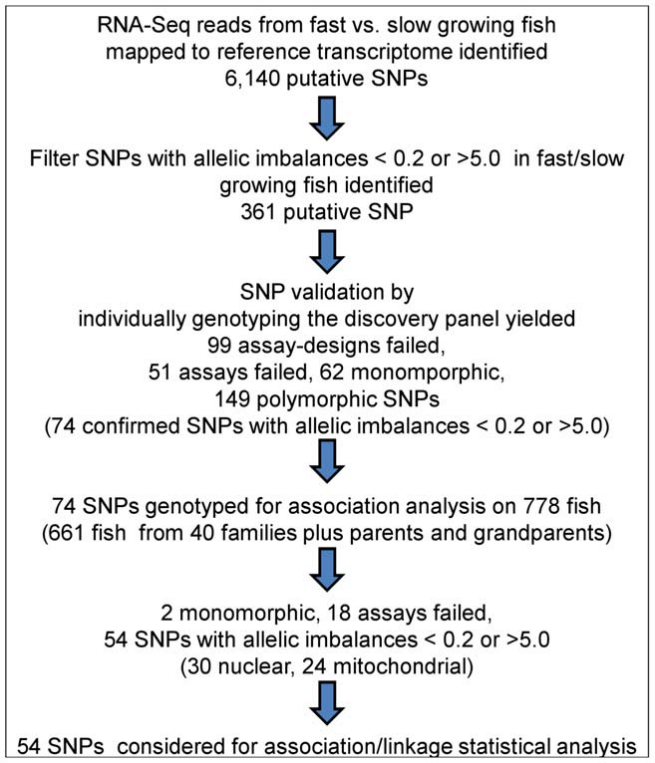
Workflow used for discovery of SNPs associated with growth traits in the rainbow trout transcriptome. SNPs identified in RNA-Seq reads were called and filtered using Alpheus pipeline. The SNP detection stringency conditions include at least 4 reads calling the variant, >20% reads calling the variant and >20 Quality score. SNP putatively associated with fast growth were considered at allelic imbalances scores >5.0 as an amplification and <0.2 as loss of heterozygosity. SNPs were validated by individually genotyping the discovery panel. Putative SNPs were genotyped for association analysis on 778 fish (40 families).

Single-end short read (36-bp) RNA-Seq technology was used to identify putative SNPs in cDNAs from fast growing versus slow growing rainbow trout fish. Sequencing one lane of an Illumina Genome Analyzer flow-cell from a pool of 10 samples from the fast growing fish yielded 8,275,289 reads; from which 6,077,479 (73%) were mapped to a transcriptome reference sequence with 1,624,444 (20%) uniquely mapped to transcripts. Similarly, sequencing a cDNA library from a pool of 10 slow growing fish on a separate flow-cell lane yielded 6,340,991 reads; from which 3,981,922 (63%) were mapped to the reference transcriptome with 1,190,804 (19%) uniquely mapped to transcripts. Figure 1 summarizes the workflow used for discovery of putative SNPs associated with growth traits in the rainbow trout transcriptome. A total of 6,140 putative SNPs were identified using SNP detection stringency conditions of at least 4 reads calling the variant, >20% reads calling the variant and >20 Quality score (Figure 1). To overcome difficulties in distinguishing true/false SNPs due to the nature of duplicated rainbow trout genome; reads were mapped to a transcriptome reference which we previously generated from a doubled haploid rainbow trout individual [16]. SNPs due to paralogous loci were removed. Allelic frequencies of heterozygous SNPs were obtained for the pooled samples by counting the number of reads representing each allele. Allele frequency ratios between the fast growing fish samples and that of the slow growing fish samples were used to calculate allelic imbalance scores. A total of 361 SNPs putatively associated with fast/slow growth were identified using allelic imbalances cut off values >5.0 as an amplification and <0.2 as loss of heterozygosity (Figure 1).

To validate heterozygosity and allelic imbalance scores of the putative SNPs, a Sequenom iPLEX platform was used to individually genotype each of the 20 fish of the SNP discovery panel. Ninety-nine SNPs failed the Sequenom iPLEX Genotyping assay design, mostly because SNP flanking sequences were less than the 100-bp long requirement for iPLEX assay design. The remaining 262 SNPs were submitted to Sequenom genotyping, of which 211 SNPs were successfully genotyped and 51 SNPs failed due to technical multiplexing errors (∼81% SNP conversion rate). Sequenom genotyping showed that 149 SNPs are heterozygous and 62 are monomorphic (Figure 1). Seventy-four SNPs proved to be polymorphic and potentially associated with growth rate by genotyping fish of the discovery panel (allelic imbalances >5.0 or <0.2) were further evaluated as growth-associated markers by genotyping a panel of 778 fish from 3 generations (Figure 1). Two SNPs turned out to be monomorphic and 18 SNPs did not yield satisfactory genotyping results due to unacceptable genotyping quality or showed abnormal allelic variation. The remaining 54 validated SNPs were considered for the final association study as explained below; 30 nuclear SNPs (nuSNPs) and 24 mitochondrial SNPs (mtSNPs) (Figure 1 and table S1).

These results indicate about 70% success rate of the pooled sequencing strategy in detecting true SNPs (147 true SNPs out 211 successful assays). Pooled sequencing is a cost-effective but challenging strategy for detection of variants, especially rare ones. Detection of SNPs from pooled sequencing requires high stringency methods and deep sequence coverage compared to individual genotyping strategy. To overcome the next generation technical sequencing error that causes false SNP discovery, individual genomes are typically sequenced to 20–30X depth of coverage [34]. In this study, although a sequence coverage of only ∼0.97X (∼0.73M read) per fish was used, approximately 70% success rate in detecting polymorphic/true SNPs was achieved. This success rate in detecting true SNPs is higher than what we previous achieved in rainbow trout (48%) using genomic reduced representation libraries and pyrosequencing technologies [35]. Higher accuracy rate of RNA-Seq in SNP discovery (81%) was reported in a bovine milk transcriptome study [36]. A lower level of accuracy in SNP detection is expected in rainbow trout due to typical issues of the genome duplication reported for salmonids including errors in assembling paralogous sequences [37], [38], [39]. Because of low sequence coverage used in this study, SNPs identified probably represent the most common variants in the populations. Further studies to detect rare variants using higher depth of coverage are warranted.

### Phenotypic Variation in Growth

**Figure 2:**
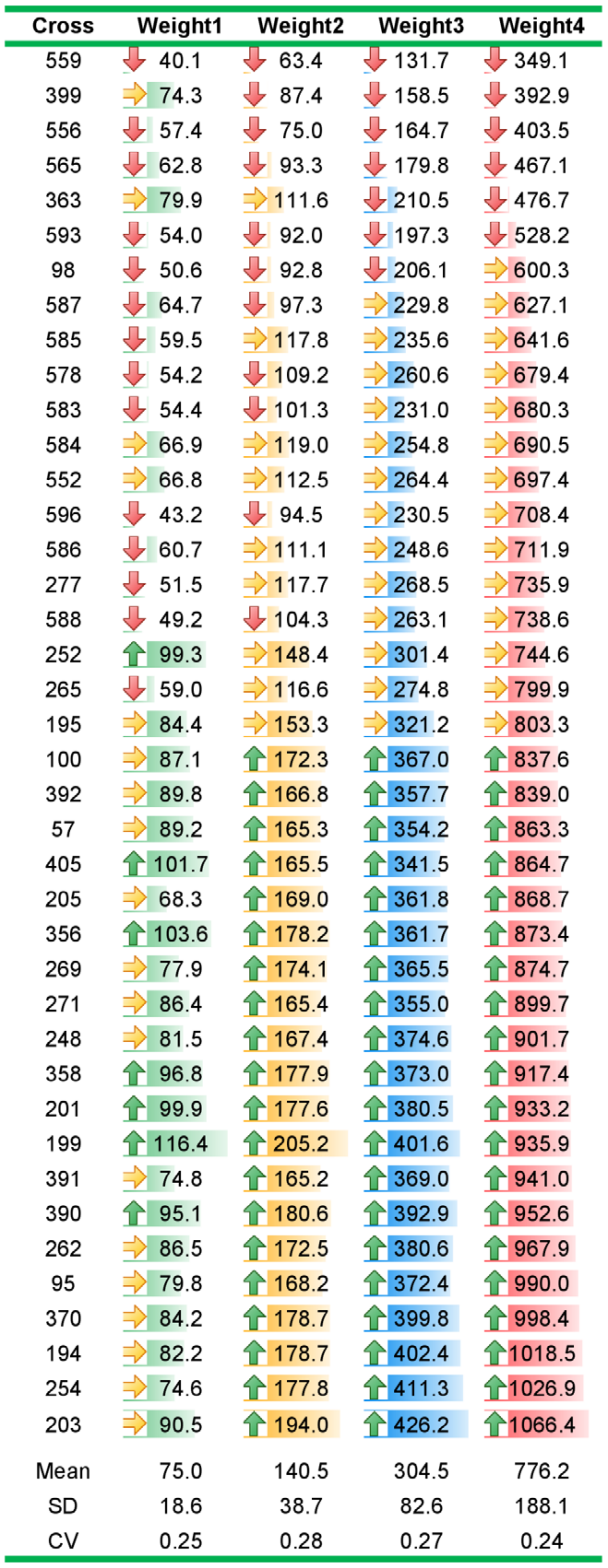
Variation in average family body weight (BW) measured in grams at approximately 6, 7, 9 and 12 months post-hatching (Weight1, Weight2, Weight3 and Weight4). CV (SD/mean) indicates the phenotypic coefficients of variation. Color intensities (green, blue and red) reflect changes in mean of BWs of different families at Weight1, Weigh2 and Weight3, respectively. Up/right/down arrows indicate families’ mean BWs lie within top, middle and bottom 33% of the population at each age, respectively.

In this study, individual BW was measured at four ages, 6, 7, 9 and 12 months post-hatching; Weight1, Weight2, Weight3 and Weight4, respectively. There was substantial variation in BW means measured at each age (Figure 2) among the 40 families used in this study. The phenotypic coefficients of variation (CV = SD/mean) in the tested population were, 25, 28, 27 and 24% for Weight1, Weight2, Weight3 and Weight4, respectively. Mean BW of the fastest growing family at 12 month was ∼305% larger than that of the slowest growing family (Figure 2). There was a positive response to selection in the whole population, genetic trend estimate for 10-month BW was 60.0 grams per generation from 2006–2010 (unpublished data).

Design variables, fixed effects and covariates contribution to the predictive power of growth trait models were assessed with stepwise model selection, and we found that family as independent variable has significant effects on Weight1 when using model A (*P*≤0.0001, Table S2). Similarly, fish age showed significant contributions to the predictive power of Weight1-4 and tank effects on the predictive power were significant at Weight2 and Weight3 (*P*<0.01, Table S2). Interestingly, the founder-strain composition effect showed that Shasta strain has significant contributions to weight at each age (Statistical model A &B, *P*<0.01, Table S2). Silverstein reported significant differences among crosses at NCCCWA in thermal growth coefficient, percent of feed consumption and residual feed intake when fish were fed to satiation. However, there was no significant difference among families, including control Shasta crosses, when feed was moderately restricted [40]. The observation of superior contribution of the Shasta founder strains to the phenotypic growth traits is an important finding for breeding activities at NCCCWA. However, this study was not optimally designed to compare strains contribution to growth traits. Shasta founder strains superiority to body weight warranted further examinations to clarify any possible issues related to unbalanced sample sizes per strain. As explained in the methods section, the random family, fixed effect tank and covariates age and founder-strain composition SH were included in the linear model of association analysis to account for these variables and ensure that the association signals were due to marker effects.

Multivariate normal distribution for residuals diagnostic tests (including Skewness and Kurtosis) revealed significant departures from normality for growth response traits Weight1 and Weight4 (Table S2). However, Weight4 has been used in quantitative trait analysis due to its relatively low Kurtosis (Kurtosis <0.54). Weight2 and Weight3 did not have a significant departure from MVN distribution (Kurtosis <0.24; Shapiro-Wilk *P*>0.01). All SGR measurements showed deviations from MVN distribution (data not shown). Therefore, only BW data from Weight2, Weight3 and Weight4 were included in association analysis.

### Markers Association with Growth

#### Nuclear SNPs association with growth

A family-based marker association analysis of 30 validated nuclear SNPs with growth traits on 778 fish (the t-statistic for regression of phenotype on allele count) was performed with PLINK program [22]. Eight markers were significantly associated with BW; nuSNP 1, 7, 9, 17, 22, 23, 24, 25 (Table 1, P<0.01). Markers nuSNP 1 and 7 were associated with BW at all 3 ages (P<0.01). All eight markers were associated with Weight2, 6 markers were associated with Weight3, and 4 markers with Weight4 (P<0.01).

**Table 1:** Association of nuclear SNPs with growth traits^1^ using family-based association analysis^2^.

SNP	Weight2				Weight3				Weight4			
	*b_y.x_*	*t*	*P*	*P_empirical_*	*b_y.x_*	*t*	*P*	*P_empirical_*	*b_y.x_*	*t*	*P*	*P_empirical_*
nuSNP1	−10.3	−3.3	0.00096**	0.096	−25.6	−2.8	0.00431**	0.112	−48.8	−2.6	0.00782**	0.084
nuSNP7	30.6	3.5	0.00049**	0.075	83.6	3.2	0.00124**	0.082	144.8	2.8	0.00468**	0.07
nuSNP9	−12.8	−2.6	0.00781**	0.141	−24.8	−1.8	0.06913	0.272	−119	−4.3	1.52e−05**	0.002*
nuSNP17	10.8	3.1	0.00179**	0.136	27.5	2.8	0.00517**	0.133	47.5	2.3	0.02046*	0.152
nuSNP22	−15.4	−3.7	0.00022**	0.058	−32.9	−2.7	0.00581**	0.116	−54.4	−2.2	0.02665*	0.144
nuSNP23	−16.9	−4.2	0.995e−05**	0.032*	−38.4	−3.3	0.00078**	0.059	−58.1	−2.3	0.01821*	0.126
nuSNP24	−15.7	−3.7	0.00018**	0.051	−33.1	−2.7	0.00576**	0.112	−54.4	−2.2	0.02721*	0.147
nuSNP25	9.4	2.6	0.00775**	0.221	22.2	2.1	0.03076*	0.258	61.2	2.7	0.00566**	0.088

1 Body weight was recorded on each animal at approximately 7 (Weight2), 9 (Weight3) and 12 (Weight4) months post-hatching.

2 Family-based association analysis was performed with program PLINK version 1.07 [22]. Here, *t* is the *t*-statistic for regression of phenotype on allele count (***by.x***), *P* is the asymptotic *P*-value for *t*-statistic, and the empirical *P*-value was estimated using 20,000 permutations.

** indicates significance at *P<*0.01.

* indicates significance at *P<*0.05.

The family-based association analysis performed with the R-package (GWAF) [23] identified 3 strongly associated markers with BW of at least 2 ages; nuSNP 7, 20, and 21 (*P* = 1.5E−19 to 9.2E−63, table 2). Two other SNPs showed weaker association with BW, nuSNP 8 & 12 (*P* = 2.0E−5 to 8.1E−09). Each SNPs, except nuSNP 12, explained <1% of phenotype variation 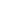.

**Table 2:** Family-based association analysis of nuclear SNPs with growth traits^1^ using the R package GWAF^2^.

SNP	Weight2					Weight3					Weight4				
	χ^2^	DF	*P*	Model^3^	h^2^_q_	χ^2^	DF	*P*	Model^3^	h^2^_q_	χ^2^	DF	*P*	Model^3^	h^2^_q_
nuSNP7	233.08	1	1.27E−52**	D	0	81.86	1	1.46E−19**	D	0	12.45	1	0.00041	D	0
nuSNP8	2.38	1	0.12291	D	0	33.25	1	8.10E−09*	D	0	0.56	1	0.4539	D	0
nuSNP12	8.43	2	0.01478	G	0.01	21.21	2	0.00002*	G	0.01	17.27	2	0.00017*	G	0.01
nuSNP20	279.6	1	9.20E−63**	D	0	101.75	1	6.31E−24**	D	0	15.91	1	6.65E−05*	D	0
nuSNP21	245.88	1	2.05E−55**	D	0	101.14	1	8.59E−24**	D	0	12.22	1	0.00047	D	0

1 Body weight was recorded on each animal at approximately 7 (Weight2), 9 (Weight3) and 12 (Weight4) months post-hatching.

2 The genome-wide association analysis with family data (GWAF) R package [23] was used in the association analysis. The analyzed sample included 40 full-sib families each with ∼17 progeny. Here, we show the asymptotic *P*-value for the test statistic distributed as a χ^2^ with 1 and 2 DF for dominant and general model, respectively. The 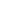 is the proportion of phenotypic variance explained by the tested SNP.

3 The general (G) and dominant (D) models had the highest likelihood in the association analysis of Weight2, Weight3 and Weight4.

** indicates SNPs strongly associated with BW (*P*-value = 1.5E−19 to 9.2E−63).

* indicates SNPs with weaker association (*P*-value = 2.0E−5 to 8.1E−09).

Family-based association analyses were conducted using three methods with SOLAR [24]; the measured genotype test, the quantitative disequilibrium test (QTDT), and the quantitative trait linkage-disequilibrium test (QTLD). All 3 tests revealed evidence for association of 4 markers with BW of at least 2 ages; nuSNP 7, 12, 21, 25 (*P*<0.05, table 3). The association tests (QTLD and QTDT) were approximately similar in the empirical estimates of SNP linkages to growth traits. The QTLD and QTDT are powerful tests in identifying association due to linkage disequilibrium [41], and they are robust to population stratification.

**Table 3:** Association of nuclear SNPs with weight^1^ using family-based quantitative trait linkage disequilibrium (QTLD) analysis, the measured genotype test and the quantitative disequilibrium test (QTDT) 2.

SNP	*P*-value (Weight2)				*P*-value (Weight3)				*P*-value (Weight4)			
	Stratifi-cation	Measured genotype	QTDT^3^	QTLD	Stratifi-cation	Measured genotype	QTDT^3^	QTLD	Stratifi-cation	Measured genotype	QTDT^3^	QTLD
nuSNP7	0.486	0.042*	0.032*	0.032*	0.639	0.030*	0.040*	0.040*	0.328	0.157	0.105	0.105
nuSNP12	0.124	0.149	0.065	0.065	0.368	0.009**	0.023*	0.023*	0.105	0.003**	0.016*	0.016*
nuSNP21	0.000**	1	0.125	0.125	0.000**	0.136	0.019*	0.019*	0.020*	0.047*	0.019*	0.019*
nuSNP25	0.111	0.029*	0.084	0.084	0.145	0.024*	0.064	0.064	0.559	0.198	0.265	0.265

1 Body weight was recorded on each animal at approximately 7 (Weight2), 9 (Weight3) and 12 (Weight4) months post-hatching.

2 Family-based QTLD analysis was performed with software SOLAR version 4.0 [24]. The sample included 40 FS families each with ∼17 progeny. Here, we show the asymptotic *P*-value for the test statistic distributed as a 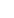 with 1 DF; the effective number of tests and multiple testing adjusted *P*-value was *P* = 0.00165 [65].

3 QTDT stands for quantitative trait disequilibrium test [21].

** indicates significance at *P<*0.01.

* indicates significance at *P<*0.05.

Family-based Bayesian quantitative trait nucleotide (BQTN) analysis was performed with software SOLAR [24] using nuclear SNP genotypes; this test is robust to multiple testing and population stratification. A single marker, nuSNP 25, had posterior probabilities 88 and 92% with Weight2 and Weight3, respectively, which indicate a significant support for a functional effect of SNP 25 on growth (data not shown). Adjusting for multiple testing showed that nuSNP 25 has significant association with Weight2 and Weight3 (*P* = 0.001, 0.0017, respectively).

Table 4 summarizes annotations and transcript locations of the aforementioned 14 markers with significant association to growth traits in rainbow trout. Significant association of 4 markers (nuSNP7, 12, 21 & 25) was detected by more than one statistical method. The average minor allele frequency of the 14 markers is 0.19. The exact *P*-value test of Hardy-Weinberg (HW) proportion for multiple alleles were simulated using PLINK program and compared to values predicted based on HW equilibrium [22]. Three markers, significantly associated with growth, exhibited significant deviation from HW nuSNP 1, 9, 17 (Table 4, HW *P*<0.01). SNPs exhibiting HW deviation are often excluded from association studies, but we maintained them because the HW deviation may be due to directional selection bias in population or partial tetraploidy genome of the rainbow trout.

**Table 4:** Summary of nuclear markers significantly associated/linked^1^ to growth traits and their annotations.

SNP	MAF^2^	HW^3^	QTDT 4 stratification test	Statistical Test(s)	Annotation^9^	Location/amino acid change
nuSNP7	0.106	NA	NA	5,6,7,8	Glucose phosphate isomerase b	5′UTR
nuSNP1	0.354	1.00E−03	NA	5	Enolase 3-1	ORF/SYN
nuSNP8	0,142	NA	NA	6	ATP2A1 calcium ATPase 3	ORF/SYN
nuSNP17	0.313	1.20E−03	NA	5	Myosin binding protein C	ORF/SYN
nuSNP20	0.033	NA	NA	6	Myosin binding protein C	ORF/SYN
nuSNP21	0.036	NA	<0.01	6,7	Myosin binding protein C	ORF/SYN
nuSNP25	0.232	NA	NA	6,7,8	Fast myotomal muscle actin 2	ORF/SYN
nuSNP22	0.199	NA	NA	5	Troponin C	3′UTR
nuSNP23	0.189	NA	NA	5	Troponin C	3′UTR
nuSNP24	0.199	NA	NA	5	Troponin C	3′UTR
nuSNP27	0.138	NA	<0.05	5	Fast myotomal muscle troponin-T-2	ORF/N→K
nuSNP29	0.235	NA	NA	5	Taxilin beta muscle-derived protein 77	ORF/G→A
nuSNP9	0.189	7.20E−03	<0.05	5	60 S ribosomal protein L4-A	ORF/SYN
nuSNP12	0.337	NA	NA	6,7	Unknown	Unknown

1 Body weight was recorded on each animal at approximately 7 (Weight2), 9 (Weight3) and 12 (Weight4) months post-hatching. A family-based sample that included 40 full-sib families each with 17 progeny were genotyped with 30 SNPs. Summary statistics were obtained with program PLINK version 1.07 [22].

2 SNPs minor allele frequency (MAF).

3 SNPs showing deviation from Hardy-Weinberg equilibrium. Exact *P*-value estimated using 20,000 permutations.

4 QTDT population stratification test.

5 *t*-statistic for regression of phenotype on allele count P is the asymptotic *P*-value for *t*-statistic, was estimated using 20,000 permutations.

6 Genome-wide association analysis with family data (GWAF) R package [23].

7 Family-based Measured Genotype and QTLD analysis was performed with software SOLAR version 4.0 [24] or QTDT quantitative trait disequilibrium test [21].

8 Family-based Bayesian quantitative trait nucleotide (BQTN) analysis was performed with software SOLAR version 4.0 [24].

9 SNP annotation; gene name and SNP location (ORF/5′UTR/3′UTR), SYN = Synonymous, NON-SYN = Non-synonymous.

NA indicates statistically insignificant estimate.

QTDT test which is especially robust to population stratification, showed that three out of the 30 evaluated nuclear SNPs has significant evidence for population stratification at *P*<0.01 and seven extra nuSNPs were significant at *P*<0.05 (Table S3), which is fairly expected in the NCCCWA closed population [42]. Only three out of the 14 markers with significant association to growth traits showed significant evidence for population stratification; nuSNP 9, 21 &27 (Table 4). However, markers association with growth traits reported here are expected to be not influenced by population stratification because we used family-based methods of association analyses, which are robust to this population stratification.

#### Biological relevance of nuSNPs to growth

Nine out of 14 nuSNP markers were located within open reading frames and 2 SNPs (nuSNP 27 &29) caused amino acid changes (Table 4). The list of growth-associated markers comprises 2 nuSNPs in genes encoding glycolytic enzymes; Glucose phosphate isomerase (GPI) and Enolase. nuSNP 7 in GPI showed strong association (measured by 4 statistical methods) with growth traits. GPI catalyzes the second step of glycolysis, conversion of glucose-6-phosphate into fructose 6-phosphate. Human mutations in GPI are characterized by anemia and neuromuscular dysfunctions [43]. An association was reported between GPI genotypes and rapid growth in the African catfish [44]. In this study, genetic variations in the glycolytic pathway genes are consistent with reports showing positive correlation of muscular glycolytic enzymes with growth rate in Atlantic cod [45] and association of increased expression of genes involved in glycolysis with selection for muscle growth [46]. In addition, we previously reported decreased transcription and translation of genes encoding glycolytic enzymes in degenerating rainbow trout muscle [47], [48].

Nine nuSNPs were identified in genes related to structural and functional muscular proteins. nuSNP 8 in ATP2A1 (calcium ATPase 3) showed association with growth. This enzyme hydrolyzes ATP to catalyze translocation of calcium from the cytosol to the sarcoplasmic reticulum. ATP2A1 is important in muscular contraction and perhaps has a role in the growth of the developing muscle [49]. In addition, the list includes 3 markers (nuSNP 17, 20 and 21) in the Myosin binding protein C transcript and 3 nuSNPs (nuSNPs 22, 23 and 24) in the Troponin C gene. Further, nuSNP25 and nuSNP27 were annotated to each of the Fast myotomal muscle actin 2 and Fast myotomal muscle troponin-T-2, respectively. Mutations in these genes may have association with muscle functions and growth but are unlikely to cause major effects on overall growth regulations.

#### Mitochondrial SNPs association with growth

Growth association of genotypes from 24 validated mitochondrial SNPs was examined using the same set of 778 fish as explained in nuSNPs. Population-based association analysis was performed with PLINK program [22]. To avoid false association signals that may arise due to individual relatedness, siblings were randomly sampled from each family to generate a population-based sample of n = 40 unrelated individuals. Random samplings were repeated to develop three sets of individuals. T-statistic test for regression of phenotype on allele count showed eight significantly associated markers with BW; mtSNP 1, 4, 6, 7, 8, 15, 16, 21 (Table 5, P<0.05).

**Table 5:** Association of mitochondrial SNPs with weight^1^ using population-based association analysis^2^.

Weight	SNP	Set1					Set2					Set3				
		*R* 2	*t*	*P*	*P_empirical_*	FDR–BH	*R* 2	*t*	*P*	*P_empirical_*	FDR–BH	*R* 2	*t*	*P*	*P_empirical_*	FDR–BH
Weight2	mtSNP6	0.05	−1.3	0.177	0.181	0.21	0.12	−2.1	0.041*	0.0417*	0.11	0.05	−1.4	0.151	0.15	0.22
	mtSNP8	0.11	−2.1	0.039*	0.039*	0.21	0.16	−2.6	0.013*	0.0134*	0.11	0.03	−1	0.302	0.3	0.31
	mtSNP21	0.11	−2.1	0.039*	0.039*	0.21	0.16	−2.6	0.013*	0.013*	0.11	0	−0.29	0.772	0.773	0.77
Weight3	mtSNP8	0.1	−2	0.052	0.053	0.34	0.09	−1.9	0.055	0.057	0.11	0.21	−3.1	0.004**	0.004**	0.05
	mtSNP21	0.02	−0.8	0.393	0.39	0.43	0.11	−1.9	0.062	0.063	0.11	0.25	−3.4	0.002**	0.002**	0.04*
Weight4	mtSNP1	0.01	−0.5	0.57	0.568	0.93	0.07	−1.6	0.106	0.105	0.18	0.12	−2	0.045*	0.044*	0.09
	mtSNP4	0.01	−0.5	0.57	0.568	0.93	0.07	−1.6	0.106	0.105	0.18	0.12	−2	0.045*	0.044*	0.09
	mtSNP7	0.01	−0.5	0.57	0.568	0.93	0.06	−1.4	0.145	0.143	0.2	0.13	−2.1	0.041*	0.040*	0.09
	mtSNP8	0.13	−2.3	0.022*	0.022*	0.53	0.22	−3.1	0.003**	0.003**	0.03*	0.12	−2.1	0.041*	0.041*	0.09
	mtSNP15	0.01	−0.5	0.57	0.568	0.93	0.07	−1.6	0.106	0.105	0.18	0.12	−2.1	0.045*	0.044*	0.09
	mtSNP16	0	−0.3	0.766	0.763	0.93	0.04	−1.3	0.192	0.191	0.2	0.14	−2.2	0.038*	0.038*	0.09
	mtSNP21	0.07	−1.6	0.114	0.113	0.93	0.27	−3.4	0.001**	0.002**	0.03*	0.13	−2.2	0.037*	0.036*	0.09

1 Body weight was recorded on each animal at approximately 7 (Weight2), 9 (Weight3) and 12 (Weight4) months post-hatching.

2 Population-based association analysis was performed with program PLINK version 1.07 [22]. From 40 full-sib families each with ∼17 progeny, a sibling was randomly sampled from each family to generate a population-based sample of *n* = 40 unrelated individuals; we repeated the random sampling to develop three sets of unrelated individuals. Here, *t* is the *t*-statistic for regression of phenotype on allele count (***by.x***); *R2* is the square of the multiple correlation coefficient which measures the proportion of total variation explained by the regression ***by.x***; *P* is the asymptotic *P*-value for *t*-statistic; the empirical *P*-value was estimated using 20,000 permutations; and FDR-BH is the false discovery rate [27].

** indicates significance at *P<*0.01.

* indicates significance at *P<*0.05.

Unlike the nuclear markers, the mitochondrial SNPs showed high *R^2^* correlation coefficient values indicating that a substantial proportion of total phenotypic variation is explained by mtSNPs (average *R^2^*/association signal = 0.16, table 5). It is worth mentioning that maternal effects have non-significant (P>0.01) contribution to the predictive power of growth traits (weight2-4) when performing both stepwise model selection [20] and quantitative genetic analysis [24] (data not presented). Haplotypes estimated for mtSNPs mapped to the mitochondrial genome [33] showed 3 distinct (24-SNP long) haplotypes (Table S4). The haplotype frequencies in the population were 0.49, 0.26 and 0.25 for Hap1, Hap2 and Hap3, respectively. *T*-statistic for regression of phenotype on haplotype count showed association of Hap3 with growth at all 3 ages (Table S4, *P*<0.05). Noteworthy, Hap2 and Hap3 share identical sequences except for mtSNP 8 and mtSNP 21. mtSNP 8 and mtSNP 21 were the most significantly associated mtSNPs and the only mtSNPs associated with BW at all 3 ages when mtSNPs were analyzed individually (Table 5). *R^2^* growth correlation coefficient values for mtSNP 8 and mtSNP 21 were 0.16 and 0.18, respectively, compared to 0.17 of Hap3. These results indicate that Hap3, mtSNP 8 and mtSNP 21 are the most significantly associated markers/haplotypes explaining phenotypic variation of growth traits in this data set.

#### Functional annotation of mtSNP markers

Eight mtSNP markers individually associated with growth traits have been identified. In addition, the 24 mtSNPs form 3 distinct haplotypes, and Hap3 was associated with growth traits. The 24 mtSNPs markers were mapped to 9 genes of the mitochondrial oxidative phosphorylation pathway (OPP) (Table 6). The OPP is an important metabolic pathway that harnesses energy released by oxidation of nutrients through catabolic biochemical processes, such as glycolysis, the citric acid cycle and beta-oxidation to produce ATP. OPP is a critical metabolic pathway in supplying the energy required for the cell, hence OPP is expected to have major effect on control of animal growth. Prevalence of the mtSNPs association with growth in rainbow trout is consistent with recent reports showing that short stature and a progressive reduction in body mass index as features of mitochondrial disease in human childhood [50], [51]. In addition, recent studies highlighted possible relationships between enhanced growth performance and mitochondrial enzyme activities in rainbow trout, catfish, broilers and livestock [52], [53], [54], [55].

**Table 6:** Summary of mitochondrial markers significantly associated 1 with growth traits and their annotations^1^.

SNP	Physical position (bp)^2^	MAF^3^	Annotation^4^	Location/amino acid change
mtSNP1	4116	0.49	NADH dehydrogenase subunit 1	ORF/SYN
mtSNP2	4323	0.49	NADH dehydrogenase subunit 1	ORF/SYN
mtSNP3	4647	0.48	NADH dehydrogenase subunit 1	ORF/SYN
mtSNP4	5212	0.48	NADH dehydrogenase subunit 2	ORF/SYN
mtSNP5	5275	0.48	NADH dehydrogenase subunit 2	ORF/SYN
mtSNP6	5530	0.48	NADH dehydrogenase subunit 2	ORF/SYN
mtSNP7	5740	0.49	NADH dehydrogenase subunit 2	ORF/SYN
mtSNP16	12423	0.47	NADH dehydrogenase subunit 4	ORF/V→ M
mtSNP17	13231	0.49	NADH dehydrogenase subunit 5	ORF/SYN
mtSNP18	13795	0.48	NADH dehydrogenase subunit 5	ORF/SYN
mtSNP19	14077	0.48	NADH dehydrogenase subunit 5	ORF/SYN
mtSNP20	14626	0.49	NADH dehydrogenase subunit 5	ORF/SYN
mtSNP21	15591	0.23	Cytochrome b	ORF/SYN
mtSNP22	15822	0.49	Cytochrome b	ORF/SYN
mtSNP23	16305	0.49	Cytochrome b	ORF/SYN
mtSNP24	16317	0.48	Cytochrome b	ORF/SYN
mtSNP8	7052	0.23	Cytochrome c oxidase subunit 1	ORF/SYN
mtSNP9	7193	0.50	Cytochrome c oxidase subunit 1	ORF/SYN
mtSNP10	8774	0.48	Cytochrome c oxidase subunit 2	ORF/SYN
mtSNP11	8804	0.48	Cytochrome c oxidase subunit 2	ORF/SYN
mtSNP14	9410	0.48	ATPase 6	ORF/SYN
mtSNP15	9656	0.49	ATPase 6	ORF/SYN
mtSNP12	9084	0.48	ATPase 8	ORF/SYN
mtSNP13	9087	0.49	ATPase 8	ORF/SYN

1 Body weight was recorded on each animal at approximately 7 (Weight2), 9 (Weight3) and 12 (Weight4) months post-hatching. Population-based association analysis was performed with program PLINK version 1.07 [22]. From 40 full-sib families each with ∼17 progeny, a sibling was randomly sampled from each family to generate a population-based sample of *n* = 40 unrelated individuals; we repeated the random sampling to develop three sets of unrelated individuals.

2 Markers were positioned on mitochondrial genome by BLASTing sequences flanking markers against a rainbow trout mitochondrial reference sequence [33].

3 SNPs minor allele frequency (MAF). ^4^SNP annotation; gene name and SNP location (ORF/5′UTR/3′UTR), SYN = Synonymous.

Twelve mtSNPs were found in 4 genes/subunits of the NADH dehydrogenase (complex I) “entry enzyme” of the OPP pathway; 3 SNPs in subunit 1 (mtSNP 1–3), 4 SNPs in subunit 2 (mtSNP 4–7), 1 SNP in subunit 4 (mtSNP 16) and 4 SNPs in subunit 5 (mtSNPs 17–20) (Table 6). mtSNP 16 in subunit 4 is the only mtSNP causing amino acid change. Existence of a large number of SNPs in complex I is consistent with human studies showing that deficiencies in complex I are the most common respiratory chain defects [56]. In addition, families with low feed efficiency showed low enzymatic activities for OPP complex I in rainbow trout tissues and broilers [53], [54].

In addition, 4 SNPs were found in the Cytochrome b gene (mtSNPs 21–24); the only subunit encoded by a mitochondrial gene of Cytochrome bc1 (complex III) (Table 6). A number of mutations in this gene have been reported in patients with myopathy suggesting an important role in muscle function [57]. In addition, muscular mitochondrial complex III showed down regulation in fish with low feed efficiency [53]. Further, 4 SNPs were found in 2 subunits of Cytochrome c oxidase (complex IV); 2 SNPs in subunit 1 (mtSNPs 8&9) and 2 SNPs in subunit 2 (mtSNP 10 &11) (Table 6). Cytochrome c oxidase has been reported to have higher activity in fast growing salmon fish [58]. Noteworthy, mtSNP 8 &21, the most significantly associated markers explaining phenotypic variation in this data set, are located in Cytochrome b and Cytochrome c oxidase, respectively.

Furthermore, 4 SNPs were found in the ATP synthase F0 (complex V), the final enzyme in the OPP pathway; 2 SNPs in subunit 6 (mtSNP 14&15) and 2 SNPs in subunit 8 (SNP 12 &13) (Table 6). These results are consistent with studies showing greater expression of the ATP synthase-α subunit of complex V in liver and lymphocytes of broilers with high feed efficiency [59]. Together, the nuSNPs and mtSNPs of the energy related genes point to importance of ATP production mechanisms in regulation of fish growth.

### Markers’ Heterozygosity in Outbred Populations

Markers’ polymorphism was assessed in other U.S. aquaculture broodstocks to evaluate the markers potential usefulness for marker assisted selective breeding in rainbow trout. Ninety-six individuals were genotyped from 12 rainbow trout broodstocks (8 fish/stock) representing 3 aquaculture populations. Forty-eight out of the fifty-four markers were polymorphic among individuals of the aquaculture populations, 3 markers were monomorphic (nuSNP 6, 29 & 30) and 3 genotyping assays failed (nuSNP 1, 8 & mtSNP 23) because of technical errors (Table S5). The average MAF of the 48 polymorphic markers on the 96 individuals was 0.24. Allelic polymorphism rate of markers was calculated in each population. The TL population had the highest number of polymorphic SNPs (47 markers, 0.26 MAF), the HF population had intermediate values (46 markers, 0.24 MAF) and CS population had the lowest polymorphism (42 markers, 0.20 MAF). Table 7 summarizes heterozygosity of the significantly associated markers to growth traits. These data indicate heterozygosity of most of the growth-associated markers in all three populations and suggest potential utility for marker assisted selection in these predominate breeds which represent most of the U.S. rainbow trout aquaculture [60]. Twenty SNPs had different minor alleles between populations, perhaps due to selection for different economic traits among the strains. Forty-two markers (out of the 51 successfully genotyped markers, 82%) were polymorphic in all 3 populations which is consistent with a report showing 71.5% average heterozygosity over nine microsatellite markers in NCCCWA founder populations [61]. Heterozygosities of most markers were confirmed in 3 outbred populations of particular importance to the aquaculture industry in the US. Therefore, these markers are suitable for MAS and population genetics studies in rainbow trout. Further studies using a larger number of phenotyped and genotyped fish are required to identify markers’ associations within each strain.

**Table 7:** Polymorphism of significantly associated/linked markers to growth traits in three aquaculture broodstocks.

SNP	TL			CS			HF		
	A1	A2	MAF	A1	A2	MAF	A1	A2	MAF
nuSNP1	Assay failed								
nuSNP7	C	A	0.02	C	A	0.03	C	A	0.03
nuSNP8	Assay failed								
nuSNP9*	A	T	0.3	T	A	0.37	T	A	0.23
nuSNP12	C	T	0.08	C	T	0.25	C	T	0.1
nuSNP17	T	C	0.38	T	C	0.1	T	C	0.06
nuSNP20	G	A	0.25	G	A	0.15	G	A	0.31
nuSNP21	G	A	0.25	G	A	0.15	G	A	0.3
nuSNP22*	G	A	0.31	A	G	0.17	A	G	0.19
nuSNP23*	C	T	0.31	T	C	0.18	T	C	0.19
nuSNP24	C	T	0.31	T	C	0.17	T	C	0.19
nuSNP25	A	G	0.13	A	G	0.35	A	G	0.16
nuSNP27	C	G	0.06	C	G	0.33	C	G	0.47
nuSNP29	Monomorphic								
mtSNP1*	G	A	0.45	A	G	0.23	G	A	0.31
mtSNP4*	A	G	0.42	A	G	0.23	G	A	0.31
mtSNP6*	T	C	0.44	T	C	0.17	C	T	0.37
mtSNP7*	G	A	0.43	A	G	0.27	A	G	0.25
mtSNP8	C	T	0.14	C	T	0.07	C	T	0.06
mtSNP15*	A	G	0.42	A	G	0.21	G	A	0.31
mtSNP16	A	G	0.45	A	G	0.23	A	G	0.25
mtSNP21	G	A	0.11	0	A	0	G	A	0.15

1 markers were genotyped in 12 broodstocks (8 unrelated fish/stock); TL (Troutlodge, Inc), CS (Clear Springs Foods) and HF (Hagerman Fish Culture Experiment Station), 48 markers were polymorphic, the average MAF is 0.24.

* indicate different minor alleles between different populations.

### Genetic/physical Mapping of SNP Markers

**Figure 3:**
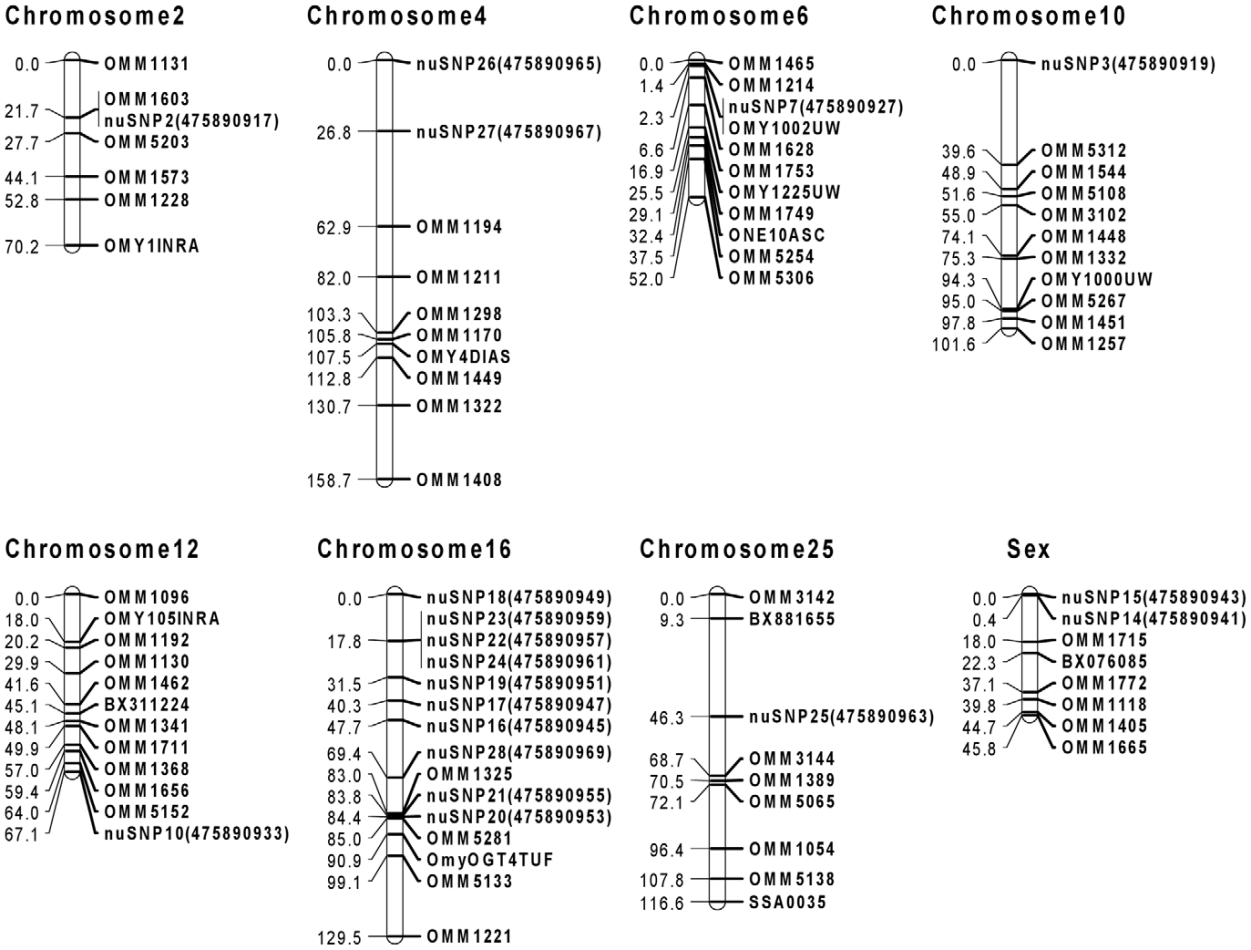
NCCCWA genetic/physical map with positions of 19 nuSNPs polymorphic markers. nuSNPs were genotyped on mapping families from the NCCCWA. Linkage groups were determined and nuSNPs were added to the NCCCWA genetic map [32]. Closest markers from the published map were determined using two-point linkage analyses.

Among the 30 nuSNPs used to genotype the four NCCCWA mapping families, 23 markers were polymorphic (Table S6). Based on the chromosomal locations of microsatellite markers on the NCCCWA reference genetic map (LOD ≥4.0) [32], 19 nuSNP markers were assigned to the chromosomes of rainbow trout (Table S6). Ten nuSNP markers were mapped to chromosome 16 (Figure 3), two nuSNP markers were placed onto each of chromosome 4 and the sex chromosome, and one nuSNP marker was assigned to each of chromosomes 2, 6, 10, 12 and 25 (Figure 3). Other polymorphic nuSNP markers could not be placed on the chromosomes of rainbow trout because they were not linked with any of the 214 microsatellite markers at LOD ≥4.

Mitochondrial markers were placed on the mitochondrial genome by BLASTing sequences flanking markers against the rainbow trout mitochondrial reference sequence (GenBank: L29771.1) [33]. Physical locations of the mtSNPs are shown in table 6.

### Conclusion

RNA-Seq approach was used in a global allele-specific expression approach to identify a set of markers associated with growth traits in rainbow trout. The study provides a proof of concept demonstrated, perhaps for the first time, in a non-model species that RNA-Seq can be used as a discovery tool; first to identify SNPs with allelic imbalances between two phenotypes, second develop genetic markers for association studies, and third to identify candidate genes explaining variations in phenotypes. In this study, only muscle tissues from two families were used as the RNA source. Other tissues, more families and life stages should be used to explore more SNPs in the population and detect tissue- or life stage-specific SNPs that are associated with growth and development. Although many of SNPs identified in this study are located outside of the open reading frames or caused synonymous mutations, marker loci may be linked to nearby other causative mutations in the genome. Further studies are needed to identify causative mutations. In addition, synonymous mutations may still have functional effects by altering the mRNA folding/stability and or by translation suppression [62], [63]. Libraries from pooled samples were sequenced at low depth of coverage, which proves that RNA-Seq is a fast, economical and effective method for marker development in species without complete genome sequences/assemblies. The RNA-Seq technique is applicable in many other species of agricultural interest that do not have complete genome sequence/assemblies or at least finished to the point where they are useful. In addition, the RNA-Seq approach we developed in this study will still be useful model species if the population diversity is high, then the numbers of genotypes/phenotypes necessary to use Whole Genome Selection is going to be so staggeringly high.

## Supporting Information

Table S1 **Summary of 54 SNPs (30 nuSNPs and 24 mtSNPs with allelic imbalances >5.0 or <0.2 in fast/slow growing fish) considered for the growth traits association study and their annotations.** SNPs were submitted to NCBI dbSNP database [64].
(DOCX)

Table S2 **Variables with significant contribution to the predictive power of growth trait1 models using stepwise model selection^2^.**
(DOCX)

Table S3 **Association of nuclear SNPs with weight^1^ using family-based quantitative trait linkage disequilibrium (QTLD) analysis^2^.**
(DOCX)

Table S4 **Association of mitochondrial SNP haplotypes with growth traits^1^ using population-based association analysis^2^.**
(DOCX)

Table S5 **Markers’ heterozygosity and minor allele frequency (MAF) in outbred populations, 96 individuals were genotyped from 12 rainbow trout families (8 fish/family) representing 3 outbred populations from the Hagerman Fish Culture Experiment Station (HF), Clear Springs Foods (CS) and Troutlodge Inc (TL).**
(XLSX)

Table S6 **30 nuSNPs were genotyped on mapping families from the NCCCWA.** Linkage groups were determined and nuSNPs were added to the a recently published version of the NCCCWA genetic map (LOD ≥ 7.0). Segregating data of nuSNPs genotypes were used to identify the closest markers from the published map using two-point linkage analyses. Linkage analysis placed 19 loci onto 8 chromosomes.
(XLSX)
