# Supplementary material for: RNA-Seq Identifies SNP Markers for Growth Traits in Rainbow Trout
Source: PLoS One. 2012 May 4;7(5):e36264. doi: 10.1371/journal.pone.0036264 (PMC3344853; doi:10.1371/journal.pone.0036264)
Supplement: Table S1 — Summary of 54 SNPs (30 nuSNPs and 24 mtSNPs with allelic imbalances >5.0 or <0.2 in fast/slow growing fish) considered for the growth traits association study and their annotations. SNPs were submitted to NCBI dbSNP database [64]. (DOCX) [file pone.0036264.s001.docx]

**Table S1.**

| **Marker** | **GenBank**  **dbSNP SS#** | **GenBank**  **BLAST**  **acc #** | **position/**  **alleles** | **Annotation** | **Fast/Slow**  **allelic imbalances** |
| --- | --- | --- | --- | --- | --- |
| nuSNP1 | 475890914 | EZ880587.1 | g164a | Enolase 3-1 | 6.84 |
| nuSNP2 | 475890917 | EZ905445.1 | c886t | GAPDH | 22.7 |
| nuSNP3 | 475890919 | EZ905496.1 | g3131a | Glycogen phosphorylase | 0.02 |
| nuSNP4 | 475890921 | EZ905496.1 | t1135g | Glycogen phosphorylase | 14.7 |
| nuSNP5 | 475890923 | EZ905668.1 | t484c | Aldolase A | 34.9 |
| nuSNP6 | 475890925 | EZ906109.1 | c593t | Triosephosphate isomerase B | 0 |
| nuSNP7 | 475890927 | EZ906266.1 | a112c | Glucose phosphate isomerase b | 7.32 |
| nuSNP8 | 475890929 | EZ907182.1 | c469t | ATP2A1_MAKNI CaATPase 3 | 35 |
| nuSNP9 | 475890931 | EZ907252.1 | t403a | 60S ribosomal protein L4-A | 0.05 |
| nuSNP10 | 475890933 | EZ914082.1 | c354t | Myosin regulatory light chain 2 | 0.01 |
| nuSNP11 | 475890935 | EZ780829.1 | a471g | Elongation factor 2-like | 39.7 |
| nuSNP12 | 475890937 | EZ783712.1 | t368c | Unknown | 0.05 |
| nuSNP13 | 475890939 | EZ765581.1 | t682g | Glycogen phosphorylase | 14.7 |
| nuSNP14 | 475890941 | EZ850783.1 | t195g | Unknown | 0.02 |
| nuSNP15 | 475890943 | EZ850482.1 | t257g | Glycogen phosphorylase | 0.02 |
| nuSNP16 | 475890945 | EZ906399.1 | g383a | ATP5C1 ATP synthase | 0.16 |
| nuSNP17 | 475890947 | EZ906429.1 | t745c | Myosin binding protein C | 0.1 |
| nuSNP18 | 475890949 | EZ764634.1 | c1345a | Phosphoglycerate mutase 2-2 | 0.01 |
| nuSNP19 | 475890951 | EZ905172.1 | g2719a | Alpha-actinin-1 | 27.4 |
| nuSNP20 | 475890953 | EZ849815.1 | a448g | Myosin binding protein C | 0.04 |
| nuSNP21 | 475890955 | EZ883107.1 | a121g | Myosin binding protein C | 0.02 |
| nuSNP22 | 475890957 | EZ764330.1 | g540a | Troponin C | 18 |
| nuSNP23 | 475890959 | EZ850118.1 | c127t | Troponin C | 10.8 |
| nuSNP24 | 475890961 | EZ764330.1 | c643t | Troponin C | 10.9 |
| nuSNP25 | 475890963 | EZ854208.1 | g542a | Fast myotomal muscle actin 2 | 0.06 |
| nuSNP26 | 475890965 | EZ764738.1 | c265g | Aspartyl beta-hydroxylase-like | 0.16 |
| nuSNP27 | 475890967 | EZ763605.1 | g310c | Fast muscle troponin-T-2 | 0.08 |
| nuSNP28 | 475890969 | EZ766104.1 | c557t | 60S ribosomal protein L10 | 0.01 |
| nuSNP29 | 475890971 | EZ765495.1 | g1219c | Taxilin beta muscle-derived 77 | 0.11 |
| nuSNP30 | 475890973 | EZ765495.1 | g1225t | Taxilin beta muscle-derived 77 | 0.13 |
| mtSNP1 | 475890975 | EZ914813.1 | a153g | NADH dehydrogenase subunit 1 | 0.03 |
| mtSNP2 | 475890977 | EZ866745.1 | g501a | NADH dehydrogenase subunit 1 | 0.06 |
| mtSNP3 | 475890980 | EZ765366.1 | g298t | NADH dehydrogenase subunit 1 | 0.1 |
| mtSNP4 | 475890982 | EZ763519.1 | a677g | NADH dehydrogenase subunit 2 | 0.12 |
| mtSNP5 | 475890984 | EZ763519.1 | a740g | NADH dehydrogenase subunit 2 | 59.6 |
| mtSNP6 | 475890986 | EZ860088.1 | t188c | NADH dehydrogenase subunit 2 | 0.08 |
| mtSNP7 | 475890988 | EZ880445.1 | g302a | NADH dehydrogenase subunit 2 | 0.01 |
| mtSNP8 | 475890990 | EZ905106.1 | t679c | Cytochrome c oxidase subunit 1 | 15.6 |
| mtSNP9 | 475890992 | EZ905106.1 | c538t | Cytochrome c oxidase subunit 1 | 0.05 |
| mtSNP10 | 475890994 | EZ906378.1 | t2202c | Cytochrome c oxidase subunit 2 | 17 |
| mtSNP11 | 475890996 | EZ906378.1 | g2172a | Cytochrome c oxidase subunit 2 | 20.9 |
| mtSNP12 | 475890998 | EZ906378.1 | t1892c | ATPase 8 | 7.66 |
| mtSNP13 | 475891000 | EZ906378.1 | a1889g | ATPase 8 | 18.7 |
| mtSNP14 | 475891002 | EZ906378.1 | a1566g | ATPase 6 | 18.9 |
| mtSNP15 | 475891004 | EZ869571.1 | a323g | ATPase 6 | 0.01 |
| mtSNP16 | 475891006 | EZ905573.1 | g560a | NADH dehydrogenase subunit 4 | 0.07 |
| mtSNP17 | 475891008 | EZ905573.1 | t1369c | NADH dehydrogenase subunit 5 | 5.3 |
| mtSNP18 | 475891010 | EZ870344.1 | c392t | NADH dehydrogenase subunit 5 | 6.59 |
| mtSNP19 | 475891012 | EZ906764.1 | a261g | NADH dehydrogenase subunit 5 | 0.06 |
| mtSNP20 | 475891014 | EZ906764.1 | t810c | NADH dehydrogenase subunit 5 | 0.12 |
| mtSNP21 | 475890912 | EZ905469.1 | a477g | Cytochrome b | 140 |
| mtSNP22 | 475891016 | EZ905469.1 | c709a | Cytochrome b | 19.5 |
| mtSNP23 | 475891018 | EZ905469.1 | t1192a | Cytochrome b | 0.08 |
| mtSNP24 | 475891020 | EZ905469.1 | g1204a | Cytochrome b | 17.3 |
