# Supplementary material for: RNA-Seq Identifies SNP Markers for Growth Traits in Rainbow Trout
Source: PLoS One. 2012 May 4;7(5):e36264. doi: 10.1371/journal.pone.0036264 (PMC3344853; doi:10.1371/journal.pone.0036264)
Supplement: Table S2 — Variables with significant contribution to the predictive power of growth trait1 models using stepwise model selection2. (DOCX) [file pone.0036264.s002.docx]

**Table S2**.

| **Model^3^** |  |  |  | | **Weight1** | |  |  | **Weight2** | |  |  | **Weight3** | |  |  | **Weight4** | |
| --- | --- | --- | --- | --- | --- | --- | --- | --- | --- | --- | --- | --- | --- | --- | --- | --- | --- | --- |
|  |  |  | ***by.x***^4^ | | ***F*** | ***Pr* > *F*** |  | ***by.x*** | ***F*** | ***Pr* > *F*** |  | ***by.x*** | ***F*** | ***Pr* > *F*** |  | ***by.x*** | ***F*** | ***Pr* > *F*** |
| **A:** | |  |  | |  |  |  |  |  |  |  |  |  |  |  |  |  |  |
| Family | |  | 0.04 | | 39.01 | <0.0001 |  |  |  | NS |  |  |  | NS |  |  |  | NS |
| SH | |  | 53.04 | | 42.44 | <0.0001 |  | 88.77 | 40.83 | <0.0001 |  | 199.18 | 39.03 | <0.0001 |  | 435.48 | 28.88 | <0.0001 |
| TL | |  |  | |  | NS^5^ |  |  |  | NS |  |  |  | NS |  |  |  | NS |
| UW | |  |  | |  | NS |  |  |  | NS |  |  |  | NS |  |  |  | NS |
| Tank | |  |  | |  | NS |  | -2.84 | 21.35 | <0.0001 |  | -1.23 | 10.58 | 0.0012 |  |  |  | NS |
| Age | |  | 1.93 | | 112.39 | <0.0001 |  | 2.20 | 203.50 | <0.0001 |  | 4.34 | 149.45 | <0.0001 |  | 8.77 | 99.09 | <0.0001 |
|  | |  |  | |  | |  |  |  | |  |  |  | |  |  |  | |
| **B:** | |  |  | |  | |  |  |  | |  |  |  | |  |  |  | |
| SH | |  | 42.62 | | 27.94 | <0.0001 |  | 88.77 | 40.83 | <0.0001 |  | 199.18 | 39.03 | <0.0001 |  | 435.48 | 28.88 | <0.0001 |
| TL | |  |  | |  |  |  |  |  |  |  |  |  |  |  |  |  |  |
| UW | |  |  | |  |  |  |  |  |  |  |  |  |  |  |  |  |  |
| Tank | |  |  | |  |  |  | -2.83 | 21.35 | <0.0001 |  | -1.23 | 10.58 | 0.0012 |  |  |  |  |
| Age | |  | 0.96 | | 100.03 | <0.0001 |  | 2.20 | 203.50 | <0.0001 |  | 4.34 | 149.45 | <0.0001 |  | 8.77 | 99.09 | <0.0001 |
|  | | | | | | |  |  |  |  |  |  |  |  |  |  |  |  |
|  | | | |  | | |  |  |  |  |  |  |  |  |  |  |  |  |
| Normality test *P*^6^ | | | | | <0.0001 | |  |  | 0.015 | |  |  | 0.174 | |  |  | <0.0001 | |
| Kurtosis | |  |  | | 8.85 | |  |  | 0.24 | |  |  | 0.11 | |  |  | 0.54 | |

^1^Body weight was recorded on each animal at approximately 6, 7, 9 and 12 months post-hatching (Weight1, Weight2, Weight3 and Weight4). In stepwise model selection, we used growth records from hatch year 2010 offspring (*n* = 1,657 animals) from the growth selected families, animals from the growth selected families with their corresponding parents and grandparents were used in the association analysis.

^2^Stepwise model selection was performed with SAS procedure REG [19] using significance level (SL) of SLENTRY = 0.50 and SLSTAY = 0.05.

^3^Model A included: design variable family, fixed effect tank and covariates (age, SH, TL and UW). Model B included: fixed effect tank and covariates (age, SH, TL and UW). Founder-strain composition effects were: SH = Shasta; TL = Troutlodge; and UW = University of Washington.

^4^Parameter estimate from stepwise model selection with SAS Procedure REG [19].

^5^NS indicates the variable had a non-significant (*P* > 0.05) contribution to the predictive power of the growth trait model.

^6^P-value from Shapiro-Wilk normality test (Departure from multivariate normal).
