# Supplementary material for: RNA-Seq Identifies SNP Markers for Growth Traits in Rainbow Trout
Source: PLoS One. 2012 May 4;7(5):e36264. doi: 10.1371/journal.pone.0036264 (PMC3344853; doi:10.1371/journal.pone.0036264)
Supplement: Table S3 — Association of nuclear SNPs with weight1 using family-based quantitative trait linkage disequilibrium (QTLD) analysis2. (DOCX) [file pone.0036264.s003.docx]

**Table S3**. Association of nuclear SNPs with weight^1^ using family-based quantitative trait linkage disequilibrium (QTLD) analysis^2^.

| **SNP** |  | ***P*-value (weight2)** | | | |  | ***P*-value (weight3)** | | | |  | ***P*-value (weight4)** | | | |
| --- | --- | --- | --- | --- | --- | --- | --- | --- | --- | --- | --- | --- | --- | --- | --- |
|  |  | **Stratification^3^** | **Measured genotype** | **QTDT^4^** | **QTLD** |  | **Stratification** | **Measured genotype** | **QTDT^4^** | **QTLD** |  | **Stratification** | **Measured genotype** | **QTDT^4^** | **QTLD** |
| snp1 |  | 0.17378 | 0.52912 | 0.80523 | 0.80523 |  | 0.23715 | 0.44513 | 0.65837 | 0.65837 |  | 0.15184 | 0.28461 | 0.48116 | 0.48120 |
| snp2 |  | 0.01923* | 0.71490 | 0.92088 | 0.92089 |  | 0.16293 | 0.87089 | 0.67652 | 0.67655 |  | 0.57230 | 0.31046 | 0.27242 | 0.27245 |
| snp3 |  | 0.66521 | 0.30118 | 0.33757 | 0.33757 |  | 0.31768 | 0.93002 | 0.35276 | 0.35283 |  | 0.64592 | 0.31073 | 0.34754 | 0.34783 |
| snp4 |  | 0.43313 | 0.91847 | 0.79903 | 0.79903 |  | 0.69062 | 0.76657 | 0.71386 | 0.71386 |  | 0.71056 | 0.79346 | 0.74415 | 0.74425 |
| snp5 |  | 0.26919 | 0.73080 | 0.99661 | 0.99661 |  | 0.48653 | 0.73783 | 0.60721 | 0.60721 |  | 0.48304 | 0.26012 | 0.20047 | 0.20047 |
| snp6 |  | 0.06686 | 1.00000 | 0.17460 | 0.17460 |  | 0.00006** | 1.00000 | 0.25897 | 0.25897 |  | 0.32950 | 1.00000 | 0.59181 | 0.60012 |
| snp7 |  | 0.48620 | 0.04238* | 0.03215* | 0.03215* |  | 0.63858 | 0.03018* | 0.03996* | 0.03996* |  | 0.32782 | 0.15744 | 0.10524 | 0.10524 |
| snp8 |  | 0.97316 | 0.85676 | 0.86403 | 0.86402 |  | 0.90948 | 0.51238 | 0.53158 | 0.53162 |  | 0.81440 | 0.70442 | 0.74023 | 0.74030 |
| snp9 |  | 0.02770* | 0.98285 | 0.50877 | 0.50877 |  | 0.03936* | 0.83390 | 0.70579 | 0.70582 |  | 0.02667* | 0.30462 | 0.69876 | 0.69880 |
| snp10 |  | 0.03474* | 0.92723 | 0.64624 | 0.64624 |  | 0.70890 | 0.91639 | 0.96799 | 0.96803 |  | 0.74939 | 0.19239 | 0.21786 | 0.21787 |
| snp11 |  | 0.44039 | 0.82110 | 0.96329 | 0.96329 |  | 0.35602 | 0.83558 | 0.68078 | 0.68084 |  | 0.08399 | 0.30301 | 0.15877 | 0.15877 |
| snp12 |  | 0.12404 | 0.14943 | 0.06502 | 0.06502 |  | 0.36838 | 0.00867** | 0.02328* | 0.02328* |  | 0.10539 | 0.00299** | 0.01578* | 0.01578* |
| snp13 |  | 0.76492 | 0.74463 | 0.80907 | 0.80909 |  | 0.45617 | 0.91364 | 0.84993 | 0.84994 |  | 0.68591 | 0.80181 | 0.88077 | 0.88079 |
| snp14 |  | 0.29456 | 0.79011 | 0.63514 | 0.63514 |  | 0.01499* | 0.55684 | 0.89530 | 0.89530 |  | 0.31809 | 0.62985 | 0.77442 | 0.77442 |
| snp15 |  | 0.05469 | 0.74307 | 0.92866 | 0.92867 |  | 0.62916 | 0.64985 | 0.58970 | 0.58970 |  | 0.49763 | 0.43008 | 0.36968 | 0.36969 |
| snp16 |  | 0.32911 | 0.32073 | 0.43332 | 0.43332 |  | 0.95306 | 0.30875 | 0.31204 | 0.31205 |  | 0.68717 | 0.21319 | 0.19464 | 0.19466 |
| snp17 |  | 0.77980 | 0.65898 | 0.73357 | 0.73357 |  | 0.81996 | 0.71490 | 0.67830 | 0.67831 |  | 0.89845 | 0.90593 | 0.88277 | 0.88279 |
| snp18 |  | 0.27809 | 0.27795 | 0.20878 | 0.20878 |  | 0.01559* | 0.83414 | 0.86463 | 0.86475 |  | 0.14002 | 0.80929 | 0.99371 | 0.99371 |
| snp19 |  | 0.18641 | 0.52239 | 0.77189 | 0.77189 |  | 0.16036 | 0.75826 | 0.98990 | 0.98990 |  | 0.96402 | 0.46382 | 0.48285 | 0.48277 |
| snp20 |  | 0.28008 | 0.78092 | 0.65363 | 0.65365 |  | 0.16424 | 0.36810 | 0.15332 | 0.15334 |  | 0.50560 | 0.06963 | 0.05836 | 0.05837 |
| snp21 |  | 1.81E-8** | 1.00000 | 0.12546 | 0.12546 |  | 0.00011** | 0.13617 | 0.01945* | 0.01945* |  | 0.02031* | 0.04732* | 0.01944* | 0.01944* |
| snp22 |  | 0.57401 | 0.57536 | 0.66865 | 0.66865 |  | 0.17464 | 0.17014 | 0.28233 | 0.28233 |  | 0.55064 | 0.55738 | 0.64722 | 0.64723 |
| snp23 |  | 0.07348 | 0.89909 | 0.60083 | 0.60086 |  | 0.35338 | 0.63555 | 0.79320 | 0.79320 |  | 0.49610 | 0.84929 | 0.73806 | 0.73807 |
| snp24 |  | 0.61184 | 0.46271 | 0.54214 | 0.54214 |  | 0.20740 | 0.12042 | 0.20402 | 0.20402 |  | 0.61489 | 0.46906 | 0.54149 | 0.54150 |
| snp25 |  | 0.11147 | 0.02940* | 0.08395 | 0.08395 |  | 0.14493 | 0.02353* | 0.06353 | 0.06353 |  | 0.55945 | 0.19833 | 0.26471 | 0.26472 |
| snp26 |  | 0.01247* | 0.72323 | 0.33734 | 0.33734 |  | 0.01850* | 0.69066 | 0.35912 | 0.35912 |  | 0.03919* | 0.23554 | 0.10469 | 0.10469 |
| snp27 |  | 0.01240* | 0.29354 | 0.69952 | 0.69952 |  | 0.00233** | 0.29485 | 0.73292 | 0.73295 |  | 0.05248 | 0.66264 | 0.99332 | 0.99332 |
| snp28 |  | 0.72448 | 0.89512 | 0.97051 | 0.97051 |  | 0.02605* | 1.00000 | 0.63753 | 0.63753 |  | 0.52069 | 0.76571 | 0.65599 | 0.65599 |
| snp29 |  | 0.18780 | 0.17759 | 0.29763 | 0.29763 |  | 0.08002 | 0.07589 | 0.15223 | 0.15223 |  | 0.54126 | 0.24429 | 0.30070 | 0.30070 |
| snp30 |  | 0.28618 | 0.59308 | 0.45813 | 0.45813 |  | 0.37476 | 0.29885 | 0.40734 | 0.40734 |  | 0.84271 | 0.74786 | 0.78103 | 0.78166 |

^1^Body weight was recorded on each animal at approximately 6 (weight1), 7 (weight2), 9 (weight3) and 12 (weight4) months post-hatching.

^2^Family-based QTLD analysis was performed with software SOLAR version 4.0 (Almasy and Blangero, 1998). The sample included 40 FS families each with ~17 progeny. Here, we show the asymptotic *P*-value for the test statistic distributed as a $\chi^{2}$ with 1 DF; the effective number of tests and multiple testing adjusted *P*-value was *P* = 0.00165 (Moskvina and Schmidt, 2008).

^3^QTDT test which is especially robust to population stratification, showed that three out of the 30 evaluated nuclear SNPs has significant evidence for population stratification at P<0.01 and seven extra nuSNPs were significant at P<0.05.

^4^QTDT stands for quantitative trait disequilibrium test (Abecasis et al., 2000).

** indicates significance at P< 0.01

* indicates significance at P< 0.05
