# Supplementary material for: RNA-Seq Identifies SNP Markers for Growth Traits in Rainbow Trout
Source: PLoS One. 2012 May 4;7(5):e36264. doi: 10.1371/journal.pone.0036264 (PMC3344853; doi:10.1371/journal.pone.0036264)
Supplement: Table S4 — Association of mitochondrial SNP haplotypes with growth traits1 using population-based association analysis2. (DOCX) [file pone.0036264.s004.docx]

**Table S3**. Association of mitochondrial SNP haplotypes with growth traits^1^ using population-based association analysis^2^.

| **Trait** | **Set^3^** | **# mSNPs** | **Haplotyp^4^e** | **Haplotype Sequence** | **Freq** | $\boldsymbol{\beta}_{\boldsymbol{y}\boldsymbol{.}\boldsymbol{x}}$ | ***t*** | ***P*** | ***P_empirical_*** | ***R^2^*** |
| --- | --- | --- | --- | --- | --- | --- | --- | --- | --- | --- |
| Weight2 | 1 | 24 | Hap1 | GATGACATTTGTAAGATCGCACAG | 0.525 | 23.5 | 1.90 | 0.177 | 0.1825 | 0.05 |
|  |  | 24 | Hap2 | AGGAGTGTCCACGGAGCTATAATA | 0.250 | 7.7 | 0.14 | 0.709 | 0.7179 | 0.00 |
|  |  | 24 | Hap3 | AGGAGTGCCCACGGAGCTATGATA | 0.225 | -42.8 | 4.61 | 0.039* | 0.039* | 0.12 |
|  | 2 | 24 | Hap1 | GATGACATTTGTAAGATCGCACAG | 0.487 | 25.0 | 2.67 | 0.112 | 0.1105 | 0.07 |
|  |  | 24 | Hap2 | AGGAGTGTCCACGGAGCTATAATA | 0.282 | 8.1 | 0.21 | 0.651 | 0.6410 | 0.01 |
|  |  | 24 | Hap3 | AGGAGTGCCCACGGAGCTATGATA | 0.231 | -45.5 | 6.81 | 0.013* | 0.015* | 0.17 |
|  | 3 | 24 | Hap1 | GATGACATTTGTAAGATCGCACAG | 0.513 | 20.1 | 1.79 | 0.190 | 0.1878 | 0.05 |
|  |  | 24 | Hap2 | AGGAGTGTCCACGGAGCTATAATA | 0.256 | -9.7 | 0.32 | 0.574 | 0.5725 | 0.01 |
|  |  | 24 | Hap3 | AGGAGTGCCCACGGAGCTATGATA | 0.231 | -17.8 | 0.95 | 0.338 | 0.3365 | 0.03 |
| Weight3 | 1 | 24 | Hap1 | GATGACATTTGTAAGATCGCACAG | 0.316 | 12.5 | 0.12 | 0.728 | 0.7237 | 0.00 |
|  |  | 24 | Hap2 | AGGAGTGTCCACGGAGCTATAATA | 0.421 | 42.0 | 1.69 | 0.202 | 0.2039 | 0.05 |
|  |  | 24 | Hap3 | AGGAGTGCCCACGGAGCTATGATA | 0.263 | -69.4 | 3.71 | 0.062 | 0.0629 | 0.10 |
|  | 2 | 24 | Hap1 | GATGACATTTGTAAGATCGCACAG | 0.487 | 65.1 | 3.39 | 0.074 | 0.0692 | 0.09 |
|  |  | 24 | Hap2 | AGGAGTGTCCACGGAGCTATAATA | 0.256 | -9.5 | 0.05 | 0.822 | 0.8127 | 0.00 |
|  |  | 24 | Hap3 | AGGAGTGCCCACGGAGCTATGATA | 0.256 | -79.8 | 3.71 | 0.062 | 0.0604 | 0.09 |
|  | 3 | 24 | Hap1 | GATGACATTTGTAAGATCGCACAG | 0.500 | 62.2 | 2.95 | 0.095 | 0.0938 | 0.08 |
|  |  | 24 | Hap2 | AGGAGTGTCCACGGAGCTATAATA | 0.263 | 29.4 | 0.49 | 0.488 | 0.4847 | 0.01 |
|  |  | 24 | Hap3 | AGGAGTGCCCACGGAGCTATGATA | 0.237 | -124.0 | 9.57 | 0.004** | 0.005** | 0.22 |
| Weight4 | 1 | 24 | Hap1 | GATGACATTTGTAAGATCGCACAG | 0.525 | 48.8 | 0.33 | 0.570 | 0.5662 | 0.01 |
|  |  | 24 | Hap2 | AGGAGTGTCCACGGAGCTATAATA | 0.250 | 156.0 | 2.57 | 0.118 | 0.1145 | 0.07 |
|  |  | 24 | Hap3 | AGGAGTGCCCACGGAGCTATGATA | 0.225 | -223.0 | 5.70 | 0.022* | 0.022* | 0.14 |
|  | 2 | 24 | Hap1 | GATGACATTTGTAAGATCGCACAG | 0.487 | 123.0 | 2.50 | 0.123 | 0.1251 | 0.07 |
|  |  | 24 | Hap2 | AGGAGTGTCCACGGAGCTATAATA | 0.308 | 60.7 | 0.48 | 0.493 | 0.4939 | 0.01 |
|  |  | 24 | Hap3 | AGGAGTGCCCACGGAGCTATGATA | 0.205 | -283.0 | 9.67 | 0.004** | 0.004** | 0.22 |
|  | 3 | 24 | Hap1 | GATGACATTTGTAAGATCGCACAG | 0.553 | 165.0 | 4.36 | 0.045* | 0.0430* | 0.12 |
|  |  | 24 | Hap2 | AGGAGTGTCCACGGAGCTATAATA | 0.237 | -36.4 | 0.14 | 0.713 | 0.7138 | 0.00 |
|  |  | 24 | Hap3 | AGGAGTGCCCACGGAGCTATGATA | 0.211 | -205.0 | 4.56 | 0.041* | 0.039* | 0.13 |

^1^Body weight was recorded on each animal at approximately 6 (Weight1), 7 (Weight2), 9 (Weight3) and 12 (Weight4) months post-hatching.

^2^Population-based association analysis was performed with program PLINK version 1.07 [24]. Here, *t* is the *t*-statistic for regression of phenotype on haplotype count (*by.x*); *R2* is the square of the multiple correlation coefficient, which measures the proportion of total variation explained by the regression$\beta_{y.x}$; *P* is the asymptotic *P*-value for *t*-statistic; and the empirical *P*-value was estimated using 10,000 permutations.

^3^From 40 FS families each with ~17 progeny, a sibling was randomly sampled from each family to generate a population-based sample of *n* = 40 unrelated individuals; we repeated the random sampling to develop three sets of unrelated individuals.

^4^Haplotypes estimated for mtSNPs mapped into mitochondrial genome [35] showed 3 distinct (24-SNP long) haplotypes. *T*-statistic for regression of phenotype on haplotype count showed growth association of Hap-3 at all 3 ages (**P* <0.05). Hap-2 and Hap-3 share identical sequences except for SNP 8 and SNP 21 (highlighted).
